# Supplementary figures and images for: A Virus-Packageable CRISPR System Identifies Host Dependency Factors Co-Opted by Multiple HIV-1 Strains
Source: mBio. 2023 Feb 6;14(1):e00009-23. doi: 10.1128/mbio.00009-23 (PMC9973025; doi:10.1128/mbio.00009-23)

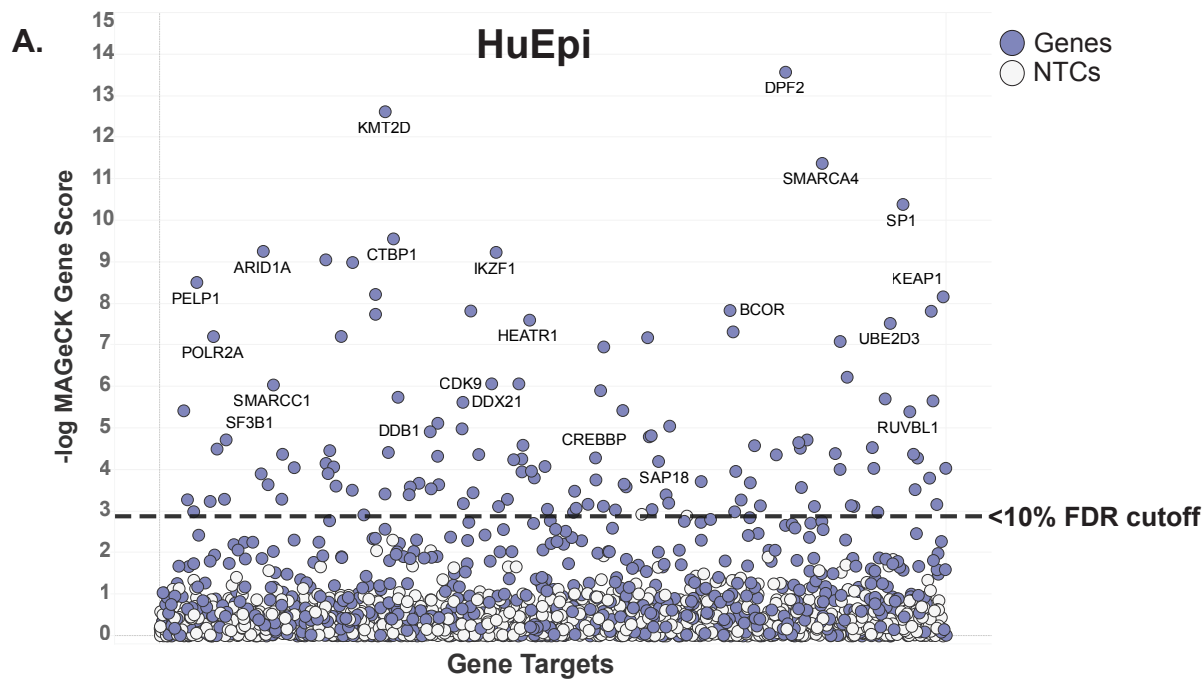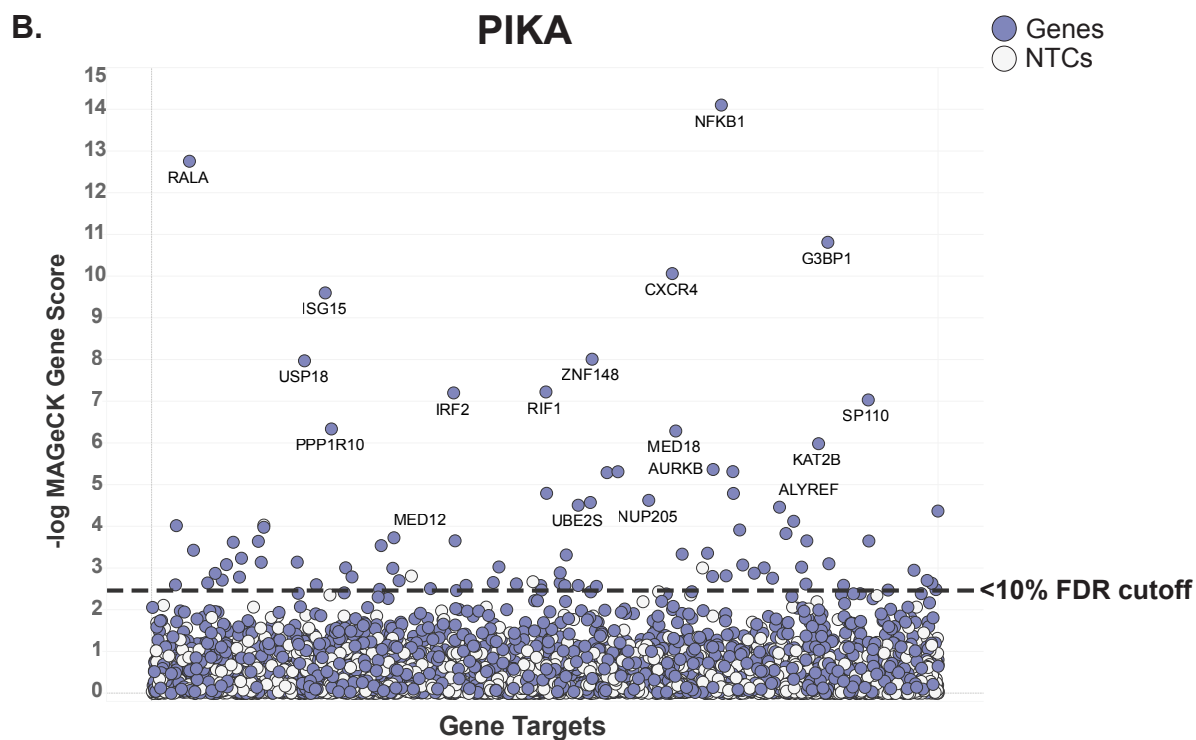

Supplement: FIG S1 [file mbio.00009-23-sf001.pdf]

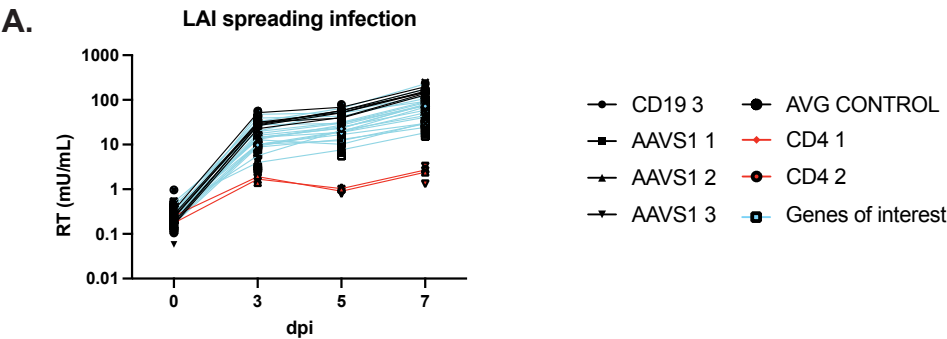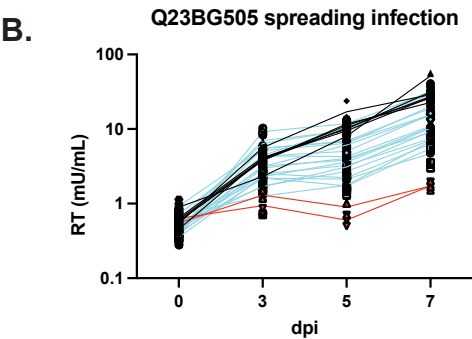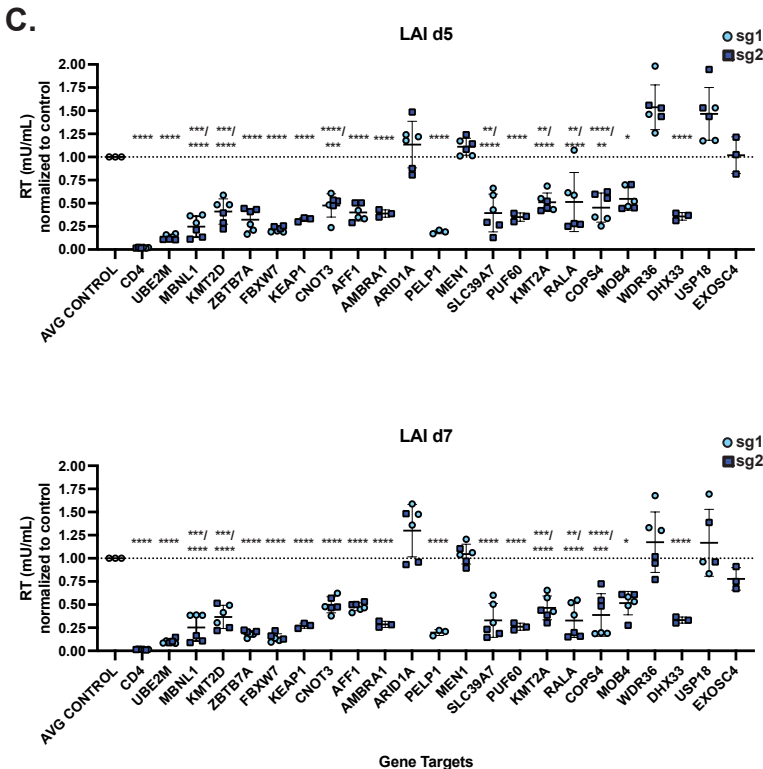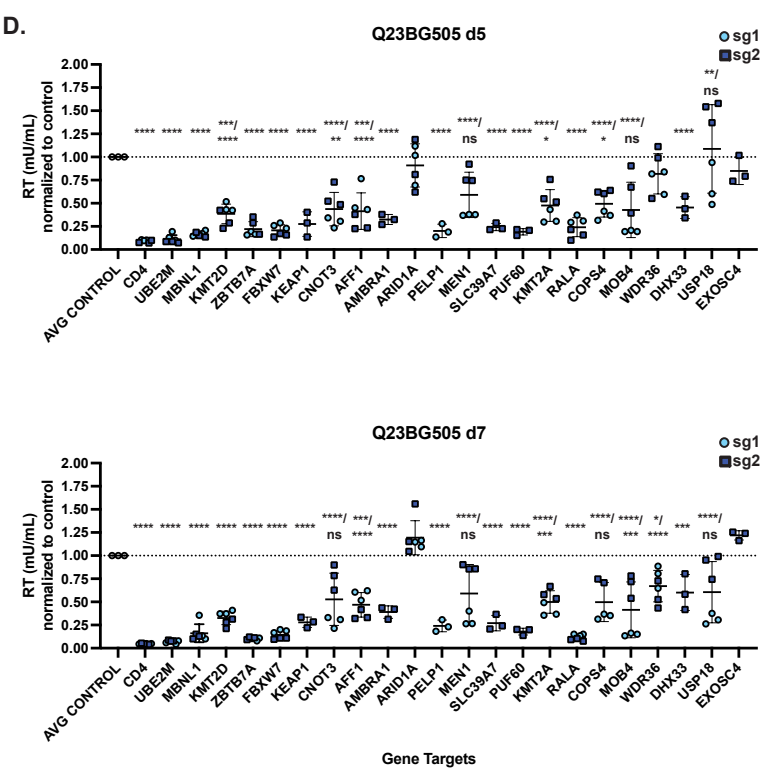

Supplement: FIG S2 [file mbio.00009-23-sf002.pdf]

A.

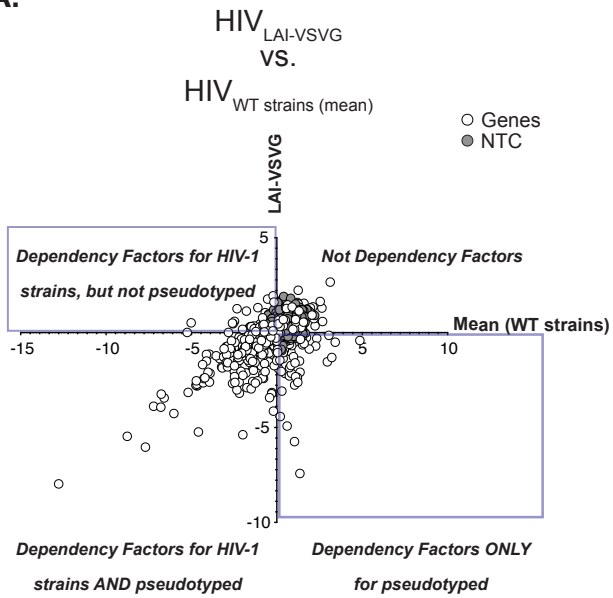

B.

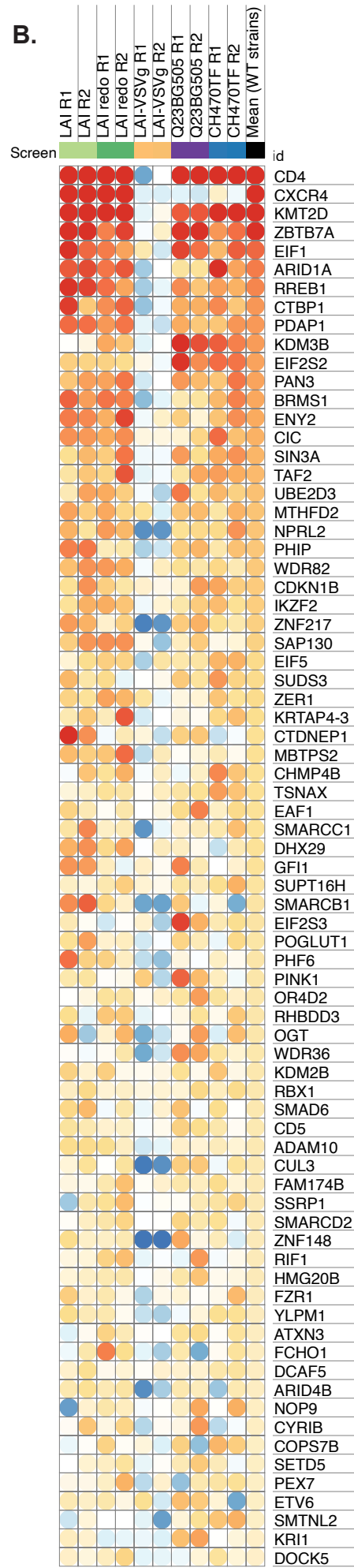

C.

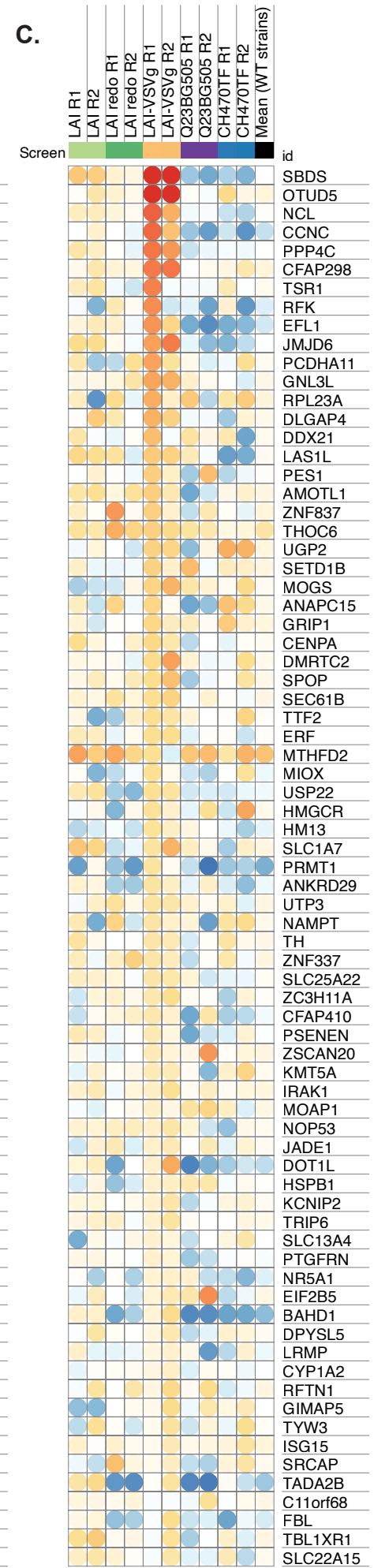

>=4 3 2 1 0 -1 -2 -3 <=-4

Supplement: FIG S3 [file mbio.00009-23-sf003.pdf]
